# Food and nutrition literacy and proactive health behavior among patients with gastric ulcer: a profile analysis of nutrition awareness and a cross-sectional mediation comparison

**DOI:** 10.3389/fpubh.2026.1926601

**Published:** 2026-09-14

**Authors:** Zhenxing Wu, Yuxin Ye, Huifeng Huang

**Affiliations:** 1 Department of Gastroenterology, Taizhou First People’s Hospital, Taizhou, China; 2 Huangyan Hospital, Wenzhou Medical University, Taizhou, China

**Keywords:** food and nutrition literacy, gastric ulcer, health behavior management, latent profile analysis, nutrition awareness, proactive health behavior

## Abstract

**Objective:**

This study aimed to explore, from both variable-centered and person-centered perspectives, the relationships among food and nutrition literacy, nutrition awareness, and proactive health behavior in patients with gastric ulcer, as well as the cross-sectional mediating role of nutrition awareness. Furthermore, this study sought to identify the latent classes of food and nutrition literacy and examine their differences in nutrition awareness and proactive health behavior.

**Methods:**

A cross-sectional study design was adopted. From March to April 2026, convenience sampling was used to recruit 418 patients with gastric ulcer from the gastroenterology outpatient and inpatient departments of two tertiary Grade A hospitals in Zhejiang Province, China.

**Results:**

Food and nutrition literacy, nutrition awareness, and proactive health behavior were significantly and positively correlated with one another. Further analysis showed that food and nutrition literacy was significantly and positively associated with both nutrition awareness and proactive health behavior. Bootstrap analysis indicated that nutrition awareness played a significant cross-sectional partial mediating role between food and nutrition literacy and proactive health behavior. Latent profile analysis showed that four latent classes were identified and labeled as the low food and nutrition literacy group, relatively low food and nutrition literacy group, relatively high food and nutrition literacy group, and high food and nutrition literacy group. Analysis of variance showed significant differences in nutrition awareness and proactive health behavior among different latent classes of food and nutrition literacy.

**Conclusion:**

In the long-term management of gastric ulcer, it is necessary to develop a stratified intervention model based on the improvement of food and nutrition literacy, with the strengthening of nutrition awareness as a key psychological pathway and latent class identification as the basis. This may provide theoretical evidence and practical references for precision nutrition education, psychological and behavioral interventions, and long-term self-management among patients.

## 1 Introduction

Gastric ulcer is a chronic disease within the spectrum of digestive disorders (1), characterized by a high prevalence, recurrent episodes, and a need for long-term management (2). Previous studies have confirmed that the occurrence and progression of gastric ulcer are not determined solely by abnormal gastric acid secretion or *Helicobacter pylori* infection (3, 4); patients’ lifestyle behaviors and psychosocial factors are also important contributors (5, 6). Although medical strategies such as proton pump inhibitors, *H. pylori* eradication therapy, and endoscopic follow-up have substantially improved the clinical outcomes of gastric ulcer (7, 8), a considerable proportion of patients still experience dietary restrictions, anxiety about recurrence, and reduced quality of life. Particularly during disease remission, patients are often required to maintain long-term health behaviors in daily life, including regular meal patterns, avoidance of irritating foods, control of tobacco and alcohol intake, symptom monitoring, and psychological stability (9, 10). Accordingly, the treatment goal for gastric ulcer has gradually shifted from lesion healing alone to patient-centered, long-term proactive health management. Proactive health behavior emphasizes the individual’s agency and sustained engagement in disease prevention, rehabilitation promotion, and health maintenance, and its level directly influences disease control, recurrence risk, and health-related quality of life (11). For patients with gastric ulcer, translating medical recommendations into stable and sustainable daily behaviors is an important pathway to improving quality of life.

Nutrition literacy refers to an individual’s comprehensive ability to access, understand, evaluate, and apply nutrition information to make appropriate dietary decisions (12, 13). It involves not only cognitive understanding of food types, meal frequency, cooking methods, and nutritional balance (14), but also the practical ability to make judgments and choices amid symptom fluctuations, work-related stress, and family dietary environments. Previous studies have found that higher nutrition literacy helps patients identify dietary risks that may impair gastric mucosal repair and understand the importance of regular eating and nutritional balance for ulcer healing and recurrence prevention (15, 16). It may also further enhance patients’ ability to communicate with healthcare professionals and participate in shared decision-making (17, 18). However, previous research has focused more on single dimensions such as nutrition knowledge or dietary adherence, which may overlook the complex process through which patients translate nutrition information into action in real-world contexts. Specifically, nutrition literacy is not a static reserve of knowledge, but rather an integrated manifestation of cognition, motivation, skills, and environmental adaptability (19). If patients have mastered basic dietary principles but lack self-regulation and situational coping abilities, their proactive health behavior may still be difficult to sustain. Therefore, examining proactive health behavior among patients with gastric ulcer from the perspective of nutrition literacy can not only extend research on behavioral mechanisms in digestive disease management, but also provide a theoretical basis for precision nutrition education and psycho-behavioral interventions. Based on this rationale, the present study attempts to answer the following two questions: (1) What is the association between nutrition literacy and proactive health behavior among patients with gastric ulcer? (2) What are the internal influencing factors underlying this relationship?

Nutrition awareness may be an important psychological factor linking food and nutrition literacy with proactive health behavior. Nutrition awareness refers to the degree of attention, value judgment, and behavioral readiness that individuals hold regarding nutrition-related health issues (20). Compared with nutrition knowledge, nutrition awareness places greater emphasis on individuals’ subjective valuation of nutrition information, risk perception, and intrinsic motivation (21, 22). According to the information–motivation–behavioral skills model (23), the formation of health behaviors depends on the joint effects of health-related information, behavioral motivation, and behavioral skills. Information alone is insufficient to ensure behavioral change; individuals also need a motivational foundation that enables them to internalize information into behavioral intention (24). Thus, food and nutrition literacy can be regarded as the competency foundation that enables patients to acquire and use nutrition information, whereas nutrition awareness is more closely aligned with the motivational component of health behavior (25). When patients with gastric ulcer have higher food and nutrition literacy, they are not only able to understand the specific requirements of dietary management, but also more fully recognize the significance of nutrition management for symptom control, ulcer healing, and recurrence prevention. This recognition may further strengthen patients’ subjective emphasis on healthy eating and their willingness for self-management, thereby promoting more proactive health behaviors.

Social cognitive theory emphasizes that individual behavior is shaped by the reciprocal interaction of cognitive, behavioral, and environmental factors, among which outcome expectations, risk perception, and self-regulation play key roles in the formation of health behaviors (26). Patients with higher food and nutrition literacy are better able to identify dietary risks, develop clearer expectations regarding the consequences of unhealthy dietary behaviors, and understand the potential benefits of healthy eating for disease management (27). These cognitive appraisals may increase patients’ sensitivity to and perceived importance of nutrition-related issues, thereby enhancing their tendency to actively adjust their diet and lifestyle. For patients with gastric ulcer, stronger nutrition awareness may make them more likely to proactively monitor their dietary behaviors in daily contexts, avoid foods that may trigger symptoms, pay attention to nutritional balance, and adjust their lifestyle or seek professional support in a timely manner when symptoms change. Therefore, nutrition awareness not only reflects patients’ attitudinal orientation toward nutrition-related issues, but may also promote proactive health behavior by enhancing risk perception, outcome expectations, and self-regulatory tendencies.

It is worth noting that the inferred relationships among nutrition literacy, nutrition awareness, and proactive health behavior are based primarily on a variable-centered perspective (28, 29). In real life, patients with gastric ulcer are not a homogeneous population; their nutrition literacy, nutrition awareness, and proactive health behavior may show substantial individual differences and distinct configurational patterns. Traditional variable-centered research usually evaluates linear relationships among variables at the overall mean level. Although this approach can reveal general associations among nutrition literacy, nutrition awareness, and health behaviors, it is difficult to identify potential heterogeneity within the patient population. Latent profile analysis can identify latent subgroups with similar psycho-behavioral patterns based on multiple observed indicators, thereby addressing the excessive reliance of traditional approaches on average group effects (30). Accordingly, from a person-centered perspective, the present study uses latent profile analysis to explore potential heterogeneity in patients’ nutrition literacy and to compare differences in nutrition awareness and proactive health behavior across different latent profiles. This approach not only allows analysis of the structural stratification of patients’ psychological resources for self-management, but also further compares differences in nutrition awareness and proactive health behavior across profiles, providing more actionable evidence for stratified interventions and precision health education.

The present study adopts complementary person-centered and variable-centered perspectives to analyze the relationships among nutrition literacy, nutrition awareness, and proactive health behavior in patients with gastric ulcer. Long-term rehabilitation in patients with gastric ulcer depends not only on pharmacological treatment and endoscopic management, but also on whether patients can develop sustained, proactive, and adaptive health behaviors in daily life. Nutrition literacy provides the cognitive foundation for patients to understand and apply dietary health information; nutrition awareness may serve as a key psychological mediator in the translation of cognition into behavior; and the heterogeneity reflected by different nutrition literacy profiles offers a new analytical perspective for explaining differences in health behavior. These findings may provide a more targeted theoretical explanation and practical basis for promoting health behaviors among patients with gastric ulcer and lay the groundwork for developing comprehensive intervention models based on nutrition literacy improvement, reinforcement of efficacy beliefs, and profile-based stratified management.

## 2 Methods

### 2.1 Study design

This study adopted a cross-sectional design to examine the relationships among nutrition literacy, nutrition awareness, and proactive health behavior in patients with gastric ulcer. It further aimed to identify latent subgroups of patients based on nutrition literacy and to compare differences in nutrition awareness and proactive health behavior across profiles.

Patients with gastric ulcer were recruited using convenience sampling from two tertiary Grade A hospitals in Zhejiang Province, China, between March and April 2026. Data were collected through questionnaire surveys. All surveys were conducted after informed consent had been obtained from participants. The study process followed the principles of voluntary participation, anonymous completion, and confidentiality of data. This study has been reviewed and approved by the Medical Ethics Committee of Taizhou First People’s Hospital.

### 2.2 Participants

#### 2.2.1 Recruitment procedure

Participants were patients diagnosed with gastric ulcer from the outpatient and inpatient departments of gastroenterology. All researchers were fully informed of the study purpose, inclusion and exclusion criteria, questionnaire distribution procedures, informed consent requirements, and ethical considerations. After patients had completed routine diagnosis and treatment or when their condition was stable, researchers conducted preliminary screening. Eligibility was determined based on electronic medical records, endoscopic findings, and clinical diagnostic information.

For patients who met the preliminary screening criteria, researchers introduced the study purpose, survey content, potential risks and benefits, principles of anonymity, and the right to withdraw through face-to-face communication. After patients signed the informed consent form, paper-based or electronic questionnaires were distributed. The questionnaires were completed independently by the patients themselves. After completion, researchers checked the questionnaires on site for missing responses, obviously patterned answers, or logical inconsistencies. If a questionnaire contained a small number of missing items, patients were asked to complete them promptly. Questionnaires with extensive missing data, continuous mechanical selection of the same response option, or missing key variables were regarded as invalid and excluded.

To enhance the accuracy of the study, strict inclusion and exclusion criteria were established. The inclusion criteria were as follows: (1) age ≥18 years; (2) a definite diagnosis of gastric ulcer confirmed by gastroscopy and clinical physicians; (3) relatively stable disease condition and sufficient physical capacity to complete the questionnaire survey; (4) basic language comprehension and communication ability; and (5) awareness of their disease diagnosis, voluntary participation in the study, and provision of written informed consent.

The exclusion criteria were as follows: (1) comorbid gastric cancer, acute gastrointestinal bleeding, perforation, pyloric obstruction, or other severe digestive complications; (2) severe dysfunction of major organs, including the heart, brain, liver, or kidneys, or a critical condition; (3) previous diagnosis of severe mental disorder, cognitive impairment, or neurological disease that prevented accurate understanding of the questionnaire content; (4) current receipt of special medical dietary therapy, enteral nutrition, or parenteral nutrition support; (5) pregnancy or lactation; and (6) missing key questionnaire variables or patterned responses that did not meet quality-control requirements.

#### 2.2.2 Minimum sample size

For the cross-sectional mediation analysis, the minimum sample size was calculated using G*Power (31). An F test was selected, and the statistical procedure was set as “Linear multiple regression: Fixed model, R^2^ deviation from zero.” The parameters were set as follows: effect size *f*^2^ = 0.15, *α* = 0.05, and 14 predictors. To achieve a statistical power of 95%, the minimum required sample size was 194 participants. For latent profile analysis, the sample size estimation was based on the large-scale Monte Carlo simulation study by Tein, Coxe (32), which indicated that when the expected number of profiles is three to five, each profile accounts for no less than 5% of the sample, and the degree of separation between profiles is moderate, a sample size of at least 300 is generally required to obtain stable and replicable classification results.

#### 2.2.3 Study sample

During the data collection period, researchers approached a total of 446 potential participants. Among them, six were younger than 18 years, and 15 had comorbidities such as gastrointestinal bleeding or pyloric obstruction. During the data cleaning stage, seven questionnaires were excluded because of missing key variables or patterned responses. Therefore, the final valid sample consisted of 418 participants, with an effective response rate of 93.72%, which met the minimum sample size requirement.

### 2.3 Measurement tools

#### 2.3.1 Food and nutrition literacy scale

Food and nutrition literacy was measured using items from the Chinese version of the Food and Nutrition Literacy Scale developed by Zhang, Zhang (33), which has been validated for cultural adaptability and reliability among Chinese adults. The scale consists of 20 items covering four dimensions: food and nutrition knowledge, access to food and planning and selecting food, preparing and making food, and eating. This scale has been widely applied in Chinese patient populations, such as patients with breast cancer (34) and patients with tuberculosis (35).

In the present study, this scale was used to assess food and nutrition literacy among Chinese patients with gastric ulcer. Items were scored on a 5-point Likert scale ranging from 1 = “strongly disagree” to 5 = “strongly agree.” The total score ranges from 20 to 100, with higher scores indicating higher levels of food and nutrition literacy among patients with gastric ulcer. In this study, the Cronbach’s alpha coefficient of the scale was 0.896.

#### 2.3.2 Nutrition awareness scale

Nutrition awareness was assessed using items from the Nutrition Awareness Scale developed by van Dillen, Hiddink (20). The scale consists of 17 items and is a unidimensional measure. In the study by Meng, Du (36), the scale was translated using the back-translation method, and confirmatory factor analysis was conducted to verify its reliability and cultural adaptability in the Chinese population. An example item is: “During the treatment of gastric ulcer, are you particular about the healthiness of the foods you eat?”

In the present study, this scale was used to measure nutrition awareness among patients with gastric ulcer. Items were scored on a 5-point Likert scale ranging from 1 = “strongly disagree” to 5 = “strongly agree.” The total score ranges from 17 to 85, with higher scores indicating higher levels of nutrition awareness among patients with gastric ulcer. In this study, the Cronbach’s alpha coefficient of the scale was 0.917.

#### 2.3.3 Proactive health behavior scale

proactive health behavior was measured using items from the proactive health behavior Scale developed by Poraj-Weder. The scale consists of 30 items and covers five dimensions: intrinsic regulation, identified and integrated regulation, introjected regulation, external regulation, and amotivation. The scale was translated into Chinese by Ge, Zheng (37), who verified its cultural reliability and adaptability in a healthy Chinese population. In addition, Wei, Zhang (38) validated its applicability in a chronic disease population with hypertension.

In the present study, this scale was used to assess proactive health behavior among patients with gastric ulcer. Items were scored on a 5-point Likert scale ranging from 1 = “completely inconsistent” to 5 = “completely consistent.” The total score ranges from 30 to 150, with higher scores indicating stronger willingness to engage in proactive health behaviors. In this study, the Cronbach’s alpha coefficient for the proactive health behavior scale was 0.928.

### 2.4 Statistical analysis

Statistical analyses were performed using SPSS version 26.0 and Mplus version 8.3. All primary study variables were analyzed using the mean item scores of the corresponding scales. First, the raw questionnaire data were organized, coded, and subjected to quality control. Invalid questionnaires with missing key variables, extensive missing responses, obvious patterned answers, or logical inconsistencies were excluded. For the final dataset included in the analysis, categorical variables were described using frequencies and percentages, while continuous variables were described using means and standard deviations. Subsequently, routine analyses were conducted, including common method bias testing, descriptive statistics and correlation analyses, skewness and kurtosis tests, and between-group comparisons.

Mediation analysis was then conducted using PROCESS Model 4 to examine the mediating role of nutrition awareness in the relationship between food and nutrition literacy and proactive health behavior. Bias-corrected bootstrap resampling was used to estimate the 95% confidence interval of the indirect effect. The proportion of the indirect effect relative to the total effect was also calculated to evaluate the relative contribution of nutrition awareness to the process by which food and nutrition literacy influences proactive health behavior. To further assess the robustness of the model, the mediation analysis was repeated without control variables, and the direction, magnitude, and significance of the total, direct, and indirect effects were compared between the model with control variables and the model without control variables.

Next, latent profile analysis was conducted using the dimension scores or item indicators of the Food and Nutrition Literacy Scale as classification indicators. Models with one to five latent classes were fitted sequentially to identify latent subgroups of food and nutrition literacy among patients with gastric ulcer. Model estimation was performed using robust maximum likelihood estimation. To avoid local optimal solutions, a large number of random starting values were specified, and the model fit indices and classification quality of different class solutions were compared. Model selection was based on a comprehensive evaluation of the Akaike information criterion, Bayesian information criterion, sample-size adjusted Bayesian information criterion, entropy, Lo–Mendell–Rubin likelihood ratio test, and bootstrap likelihood ratio test. In addition, the sample proportion of each latent class, average posterior classification probabilities, and theoretical interpretability of the classes were considered when determining the final model, in order to avoid selecting a model with superior fit indices but excessively small classes or limited clinical interpretability.

Subsequently, latent profile membership was used as the grouping variable, and one-way analysis of variance was performed to compare differences in nutrition awareness and proactive health behavior among patients with different food and nutrition literacy profiles, thereby examining the external validity of the latent profiles.

#### 2.4.1 Analytic framing of the mediation model

Because all variables were assessed at a single time point, the mediation model tested in this study should be understood as a theory-informed statistical decomposition of concurrent associations, not as an estimate of a causal mechanism. Three identification requirements for causal mediation were not met by the present design: (i) food and nutrition literacy, nutrition awareness and health behavior motivation were measured simultaneously, so the model imposes rather than tests an ordering; (ii) unmeasured dispositional variables could generate the observed nutrition awareness–motivation association; and (iii) absence of reciprocal or reverse pathways.

## 3 Results

### 3.1 Demographic characteristics

A total of 418 patients with gastric ulcer were included in this study. The mean age of the participants was 40.200 ± 9.992 years, indicating that the sample was mainly composed of young and middle-aged adults. The sex distribution was relatively balanced, with 197 male participants (47.1%) and 221 female participants (52.9%). Most participants had an educational level of junior high school to senior high school (48.6%). Detailed demographic characteristics are presented in Table 1.

**Table 1:** Demographic characteristics of the participants.

Variables	Items	Frequency (*N*)	Proportion (%)
Gender	Male	197	47.1
	Female	221	52.9
Educational level	Primary school and below	137	32.8
	Middle school–High school	203	48.6
	Bachelor degree or above	78	18.7
Place of residence	City	241	57.7
	Rural area	177	42.3
Marital status	Married	231	55.3
	Unmarried	109	26.1
	Divorced	47	11.2
	Widowed	31	7.4
Family monthly income level	4,000￥and below	125	29.9
	4,001–8,000￥	225	53.8
	8,001￥and above	68	16.3
Status of occupation	On the job	337	80.6
	Retirement	2	0.5
	Unemployed	79	18.9
Whether they had been infected with *Helicobacter pylori*^a^	Yes	143	34.2
	No	221	52.9
	Unclear	54	12.9
Smoking behavior	Yes	11	2.6
	No	407	97.4
Drinking alcohol	Yes	217	51.9
	No	201	48.1
Number of relapses/acute episodes^b^	1 time or less	150	35.9
	2 times	131	31.3
	Three times or more	137	32.8
Age (Year)	M ± SD	40.200 ± 9.992	

^a^Have you been infected with Helicobacter pylori in the past month? ^b^How many times have you experienced recurrent or acute gastric ulcer attacks in the last year?

### 3.2 Common method Bias analysis

Because this study used self-report questionnaires to collect data on food and nutrition literacy, nutrition awareness, and proactive health behavior among patients with gastric ulcer, common method bias may have been present. First, during questionnaire design, the researchers adopted anonymity and confidentiality assurance to reduce response bias caused by social desirability and evaluation apprehension.

Meanwhile, Harman’s single-factor test was used to statistically evaluate common method bias. The results showed that unrotated exploratory factor analysis extracted nine factors with eigenvalues greater than 1. The variance explained by the first common factor was 25.344%, which was below the critical threshold of 40%. Therefore, the data in this study were unlikely to be affected by serious common method bias.

### 3.3 Descriptive statistics and correlation analysis

Descriptive statistics and correlation analyses were conducted for the core study variables, as shown in Table 2. The mean scores of the core variables ranged from 3.740 to 3.763, all of which were above the medium level. This suggests that most patients with gastric ulcer had a certain degree of ability to access, understand, and apply nutrition knowledge, and also showed relatively positive nutrition awareness and health behavior tendencies.

**Table 2:** Descriptive statistics and correlation analysis of core study variables.

Variables	M	SD	Skewness	Kurtosis	1	2	3
1. Food and nutrition literacy	3.741	0.648	−1.267	0.937	1		
2. Nutrition awareness	3.763	0.733	−1.273	0.521	0.537**	1	
3. Proactive health behavior	3.740	0.640	−1.354	1.240	0.625**	0.513**	1

^**^p < 0.01.

The skewness values of the core variables ranged from −1.354 to −1.267, and the kurtosis values ranged from 0.521 to 1.240. According to the criteria proposed by Kline (39), the absolute values of skewness and kurtosis in this study were within acceptable ranges. Therefore, the data distribution did not seriously deviate from normality, allowing further correlation analysis and regression model testing.

Correlation analysis showed that food and nutrition literacy was significantly and positively correlated with nutrition awareness (*r* = 0.537, *p* < 0.01). Food and nutrition literacy was also significantly and positively correlated with proactive health behavior (*r* = 0.625, *p* < 0.01). Nutrition awareness was likewise significantly and positively correlated with proactive health behavior (*r* = 0.513, *p* < 0.01). These findings indicate that patients with gastric ulcer who had higher levels of food and nutrition literacy also tended to have stronger nutrition awareness and higher levels of proactive health behavior.

### 3.4 Between-group differences in proactive health behavior by demographic characteristics

To identify potential sociodemographic and disease-related factors associated with proactive health behavior among patients with gastric ulcer, independent-samples t tests or one-way analysis of variance were further conducted to compare differences in proactive health behavior scores across patients with different characteristics, as shown in Table 3. The results showed no statistically significant differences in proactive health behavior scores by sex, educational level, place of residence, marital status, *H. pylori* infection status, smoking behavior, alcohol consumption, or number of recurrences or acute episodes (*p* > 0.05).

**Table 3:** Between-group analysis of proactive health behavior by demographic characteristics.

Variables	Items	M	SD	*t*/*F*	*p*	Cohen d/η^2^
Gender	Male	3.794	0.610	1.624	0.105	0.159
	Female	3.692	0.664			
Educational level	Primary school and below	3.755	0.611	0.083	0.920	0.001
	Middle school - High school	3.727	0.677			
	Bachelor degree or above	3.745	0.599			
Place of residence	City	3.727	0.671	−0.482	0.63	−0.048
	Rural area	3.757	0.598			
Marital status	Married	3.734	0.634	0.761	0.517	0.005
	Unmarried	3.742	0.676			
	Divorced	3.666	0.688			
	Widowed	3.887	0.472			
Family monthly income level	4,000￥and below	3.630	0.680	3.755	0.024	0.018
	4,001–8,000￥	3.817	0.611			
	8,001￥and above	3.685	0.638			
Status of occupation	On the job	3.732	0.646	3.713	0.025	0.018
	Retirement	2.583	0.024			
	Unemployed	3.803	0.597			
Whether they had been infected with *Helicobacter pylori*^a^	Yes	3.683	0.646	0.855	0.426	0.004
	No	3.771	0.611			
	Unclear	3.760	0.738			
Smoking behavior	Yes	3.858	0.986	0.618	0.537	0.189
	No	3.737	0.630			
Drinking alcohol	Yes	3.745	0.631	0.163	0.871	0.016
	No	3.734	0.652			
Number of relapses/acute episodes^b^	1 time or less	3.749	0.635	1.135	0.322	0.005
	2 times	3.675	0.670			
	Three times or more	3.792	0.616			

^a^Have you been infected with Helicobacter pylori in the past month? ^b^How many times have you experienced recurrent or acute gastric ulcer attacks in the last year?

Notably, statistically significant between-group differences in proactive health behavior were observed for monthly household income and occupational status. Proactive health behavior scores differed significantly across monthly household income groups (*F* = 3.755 *p* = 0.024, η^2^ = 0.018). Patients with a monthly household income of 4,001–8,000 yuan had the highest proactive health behavior scores, whereas those with a monthly household income of 4,000 yuan or below had the lowest scores. This finding suggests that household economic resources may be associated with patients’ levels of proactive health behavior. However, the effect size was small, indicating that income level explained only a limited proportion of the variance in proactive health behavior.

Regarding occupational status, proactive health behavior scores differed significantly among patients with different occupational statuses (*F* = 3.713, *p* = 0.025, η^2^ = 0.018). However, only two participants were retired, resulting in an extremely small subgroup sample size and limited stability and representativeness of the mean value. Therefore, although occupational status showed a statistically significant difference, this result should be interpreted with caution.

### 3.5 Mediation analysis of nutrition awareness

Based on the results of the between-group analyses, monthly household income and occupational status were included as control variables in the mediation model to reduce the potential influence of confounding factors on the estimated relationships among the core variables. Food and nutrition literacy was entered as the independent variable, nutrition awareness as the mediator, and proactive health behavior as the dependent variable. PROCESS Model 4 was used to test the mediating role of nutrition awareness, as shown in Table 4 and Figure 1.

**Table 4:** Regression coefficients for the mediation analysis of nutrition awareness.

Independent variable	Model 1				Model 2			
	Nutrition awareness				Proactive health behavior			
	B	t	LLCI	ULCI	B	t	LLCI	ULCI
Food and nutrition literacy	0.610	13.034	0.518	0.702	0.492	11.394	0.407	0.577
Nutrition awareness					0.213	5.566	0.138	0.288
*R*	0.540					0.665		
*R* ^2^	0.292					0.442		
*F*	56.830***					81.638***		

**Figure 1:**
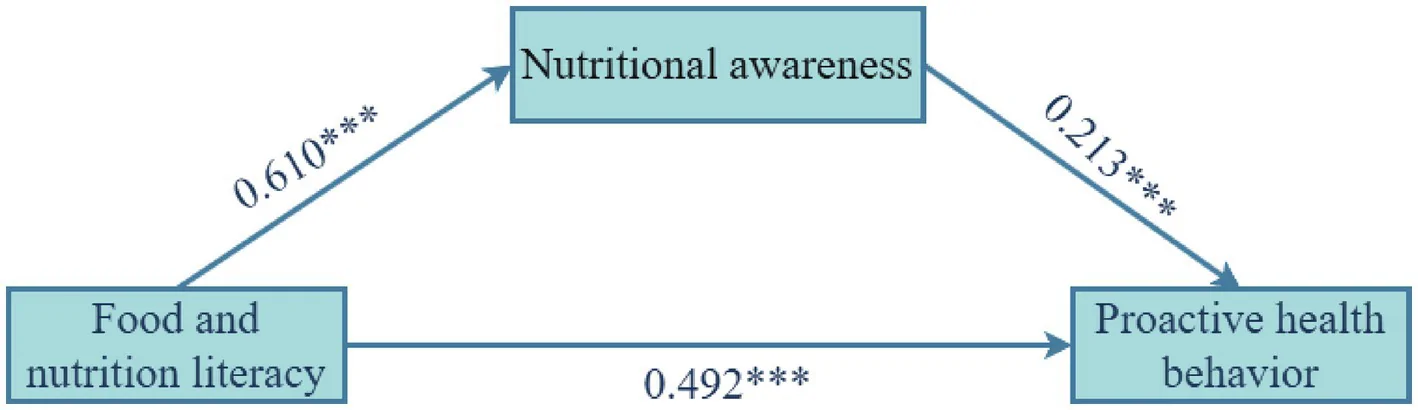
Regression coefficient plots for mediation analyses of nutrition awareness. ^***^p < 0.001.

In Model 1, food and nutrition literacy significantly and positively predicted nutrition awareness (*B* = 0.610, *t* = 13.034, 95% CI = [0.518, 0.702]). The correlation coefficient of the model was *R* = 0.540, and the coefficient of determination was *R*^2^ = 0.292. The overall model was significant (*F* = 56.830, *p* < 0.001). This result indicates that, after controlling for income and occupational status, patients with gastric ulcer who had higher food and nutrition literacy showed stronger nutrition awareness.

In Model 2, food and nutrition literacy significantly and positively predicted proactive health behavior (*B* = 0.492, *t* = 11.394, 95% CI = [0.407, 0.577]). Nutrition awareness also significantly and positively predicted proactive health behavior (*B* = 0.213, *t* = 5.566, 95% CI = [0.138, 0.288]). The correlation coefficient of the model was *R* = 0.665, and the coefficient of determination was *R*^2^ = 0.442. The overall model was significant (*F* = 81.638, *p* < 0.001). Together, food and nutrition literacy and nutrition awareness explained 44.2% of the variance in proactive health behavior among patients with gastric ulcer, indicating that both are important psycho-behavioral resources for patients’ proactive health behaviors.

The results of the bootstrap test for the mediating effect are shown in Table 5. The total effect of food and nutrition literacy on proactive health behavior was 0.618 (SE = 0.038, 95% CI = [0.543, 0.692]). The direct effect of food and nutrition literacy on proactive health behavior was 0.485 (SE = 0.043, 95% CI = [0.400, 0.570]), and the indirect effect through nutrition awareness was 0.132 (SE = 0.033, 95% CI = [0.074, 0.200]). Therefore, nutrition awareness played a significant mediating role in the relationship between food and nutrition literacy and proactive health behavior.

**Table 5:** Bootstrap test of the mediating effect.

Type of effect	Effect	SE	LLCI	ULCI	Effect proportion
Total effect	0.618	0.038	0.543	0.692	100%
Direct effect	0.485	0.043	0.400	0.570	78.58%
Indirect effect	0.132	0.033	0.074	0.200	21.42%

Further calculation of the effect proportions showed that the direct effect accounted for 78.58% of the total effect, whereas the indirect effect accounted for 21.42%. This indicates that food and nutrition literacy not only directly promotes proactive health behavior among patients with gastric ulcer, but also indirectly enhances proactive health behavior by improving nutrition awareness.

Nutrition awareness showed only a partial mediating effect, suggesting that the influence of food and nutrition literacy on proactive health behavior does not depend entirely on the improvement of nutrition awareness. In addition to nutrition awareness, food and nutrition literacy may directly affect proactive health behavior through pathways such as enhancing patients’ disease management ability, dietary decision-making ability, risk identification ability, and communication with healthcare professionals. Nevertheless, the significant mediating role of nutrition awareness suggests that simply improving patients’ nutrition knowledge may be insufficient to fully translate into sustained behavioral change. Promoting patients’ active attention to and internal recognition of the value of nutrition management is an important psychological factor in facilitating the formation of health behaviors among patients with gastric ulcer.

### 3.6 Robustness test of the mediation analysis without control variables

To further examine the robustness of the mediation model, the mediation effect analysis was repeated without including control variables, as shown in Table 6. The results showed that the total effect of food and nutrition literacy on proactive health behavior was 0.622 (SE = 0.038, 95% CI = [0.548, 0.696]). The direct effect of food and nutrition literacy on proactive health behavior was 0.492 (SE = 0.043, 95% CI = [0.407, 0.577]), and the indirect effect of nutrition awareness was 0.130 (SE = 0.033, 95% CI = [0.068, 0.196]). The confidence interval for the indirect effect also did not include zero, indicating that the mediating effect of nutrition awareness remained significant when sociodemographic variables were not controlled.

**Table 6:** Bootstrap test of the mediating effect without control variables.

Type of effect	Effect	SE	LLCI	ULCI	Effect proportion
Total effect	0.622	0.038	0.548	0.696	100%
Direct effect	0.492	0.043	0.407	0.577	79.14%
Indirect effect	0.130	0.033	0.068	0.196	20.86%

In the model without control variables, the direct effect accounted for 79.14% of the total effect, whereas the indirect effect accounted for 20.86%. These results were highly consistent with the model controlling for monthly household income and occupational status, suggesting that the relationships among the core variables did not change substantially with or without control variables. The magnitude of the total, direct, and indirect effects was similar across the two models, further supporting the stable partial mediating role of nutrition awareness in the relationship between food and nutrition literacy and proactive health behavior. This robustness test strengthens the credibility of the mediation model findings and suggests that the pathway of food and nutrition literacy–nutrition awareness–proactive health behavior has good statistical stability.

### 3.7 Latent profile analysis of food and nutrition literacy

To further identify potential heterogeneity in food and nutrition literacy among patients with gastric ulcer, latent profile analysis was conducted using food and nutrition literacy indicators as classification variables. Models with one to five classes were fitted sequentially, and model fit indicators, including AIC, BIC, aBIC, entropy, the Lo–Mendell–Rubin test, the bootstrap likelihood ratio test, and the sample proportion of each class, were comprehensively compared to determine the optimal classification model, as shown in Table 7.

**Table 7:** Fit indices for latent profile models of food and nutrition literacy (dimension of variables).

Index	Profile 1	Profile 2	Profile 3	Profile 4	Profile 5
AIC	4214.318	3437.454	3266.386	2932.866	2852.504
BIC	4246.602	3489.915	3339.025	3025.682	2965.497
aBIC	4221.216	3448.663	3281.906	2952.696	2876.645
Entropy	—	0.976	0.975	0.991	0.981
LMR (p)	—	<0.001	0.003	<0.001	0.136
BLRT (p)	—	<0.001	<0.001	<0.001	<0.001
Class 1	—	20.34%	10.53%	10.29%	9.81%
Class 2	—	79.67%	18.66%	10.77%	11.01%
Class 3	—	—	70.81%	8.61%	8.85%
Class 4	—	—	—	70.34%	61.72%
Class 5	—	—	—	—	8.61%

The model fitting results showed that as the number of classes increased from one to five, AIC, BIC, and aBIC generally decreased, indicating continuous improvement in model fit. The AIC, BIC, and aBIC values of the four-class model were markedly lower than those of the one-, two-, and three-class models. Although the AIC, BIC, and aBIC values further decreased in the five-class model, the LMR test for the five-class model was not statistically significant (*p* = 0.136), suggesting that increasing the number of classes from four to five did not significantly improve model fit. In addition, some classes in the five-class model had relatively small sample proportions, which may weaken classification stability and practical interpretability.

By contrast, the entropy value of the four-class model was 0.991, close to 1, indicating extremely high classification accuracy. The LMR test was significant (*p* < 0.001), and the BLRT was also significant (*p* < 0.001), indicating that the four-class model provided a significant improvement over the three-class model. Meanwhile, the proportions of the four classes were 10.29, 10.77, 8.61, and 70.34%, respectively, with no extremely small class. The four-class solution therefore showed good statistical stability and theoretical interpretability. Accordingly, the four-class model was selected as the optimal model for the latent profiles of food and nutrition literacy among patients with gastric ulcer.

As shown in Table 8, further examination of the average posterior classification probabilities of the four-class model showed that the average probabilities of individuals being assigned to their corresponding classes were 0.987, 0.977, 0.997, and 0.999, respectively, all substantially higher than the recommended criterion of 0.70. This indicates that the four-class model had good classification reliability. The probabilities of misclassification across classes were low, suggesting clear differentiation among the different food and nutrition literacy profiles.

**Table 8:** Average posterior classification probabilities of the four-class model.

Class	Class 1	Class 2	Class 3	Class 4
Class 1	0.987	0.013	0.001	0.000
Class 2	0.003	0.977	0.018	0.002
Class 3	0.000	0.000	0.997	0.003
Class 4	0.000	0.001	0.000	0.999

Test of robustness. Latent analysis was conducted with food and nutrition literacy measurement topics as indicator variables, as shown in Table 9, and four types of latent profiles were verified as the optimal latent profiles (AIC = 21795.528; BIC = 22211.182, aBIC = 21884.334, Entropy = 0.997).

**Table 9:** Fit indices for latent profile models of food and nutrition literacy (measure items of variables).

Index	Profile 1	Profile 2	Profile 3	Profile 4	Profile 5
AIC	25646.184	23331.780	22506.169	21795.528	21110.459
BIC	25807.604	23577.944	22837.079	22211.182	21610.858
aBIC	25680.672	23384.374	22576.87	21884.334	21217.371
Entropy	—	0.996	0.997	0.997	0.997
LMR (p)	—	<0.001	0.0002	<0.001	0.002
BLRT (p)	—	<0.001	<0.001	<0.001	<0.001
Class 1	—	19.62%	19.38%	10.77%	10.05%
Class 2	—	80.38%	11.48%	10.29%	10.77%
Class 3	—	—	69.14%	8.61%	8.13%
Class 4	—	—	—	70.34%	9.57%
Class 5	—	—	—	—	61.48%

Next, Origin 2026 software was used to plot the latent profiles of food and nutrition literacy, as shown in Figure 2. Based on the characteristics of food and nutrition literacy levels across the different classes and their subsequent performance in nutrition awareness and proactive health behavior, the four latent classes were named as follows: Class 1, “low food and nutrition literacy group”; Class 2, “moderately low food and nutrition literacy group”; Class 3, “moderately high food and nutrition literacy group”; and Class 4, “high food and nutrition literacy group.”

**Figure 2:**
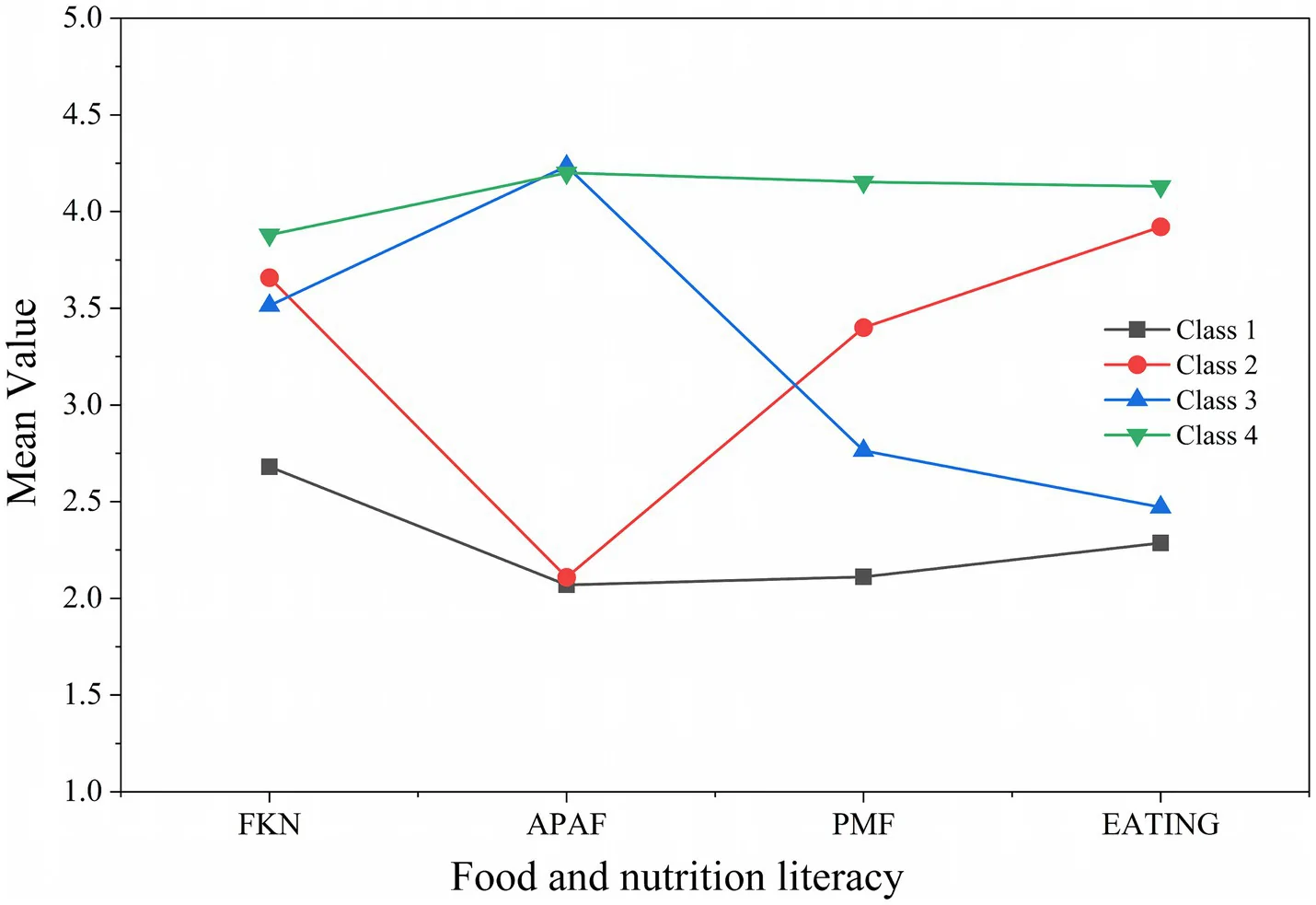
Outline of the four potential categories of food nutrition literacy. Class 1 = low food and nutrition literacy group; Class 2 = moderately low food and nutrition literacy group; Class 3 = moderately high food and nutrition literacy group; Class 4 = high food and nutrition literacy group.

Among these, the high food and nutrition literacy group accounted for the largest proportion, approximately 70.34%, indicating that most patients with gastric ulcer had relatively high levels of food and nutrition literacy. The low, moderately low, and moderately high food and nutrition literacy groups accounted for 10.29, 10.77, and 8.61%, respectively. These findings suggest that nearly 30% of patients still had varying degrees of insufficiency or uneven development in food and nutrition literacy, warranting focused attention in clinical health education.

### 3.8 Differences in proactive health behavior and nutrition awareness across the optimal latent profiles

To further verify the external validity of the latent profiles of food and nutrition literacy and compare differences in nutrition awareness and proactive health behavior among patients with different profiles, one-way analysis of variance was conducted using the four latent classes as the grouping variable and nutrition awareness and proactive health behavior as outcome variables, as shown in Table 10 and Figure 3. The results showed statistically significant differences across food and nutrition literacy profiles in both nutrition awareness (*F* = 51.258, η^2^ = 0.271, *p* < 0.001) and proactive health behavior (*F* = 78.914, η^2^ = 0.364, *p* < 0.001).

**Table 10:** Differences in proactive health behavior and nutrition awareness across the optimal latent profiles.

Class	Nutrition awareness		Proactive health behavior	
	M	SD	M	SD
Class 1	2.763	0.889	2.687	0.798
Class 2	3.468	0.793	3.461	0.699
Class 3	3.613	0.828	3.763	0.641
Class 4	3.973	0.517	3.934	0.397
*F*	51.258		78.914	
*p*	<0.001		<0.001	
η^2^	0.271		0.364	

**Figure 3:**
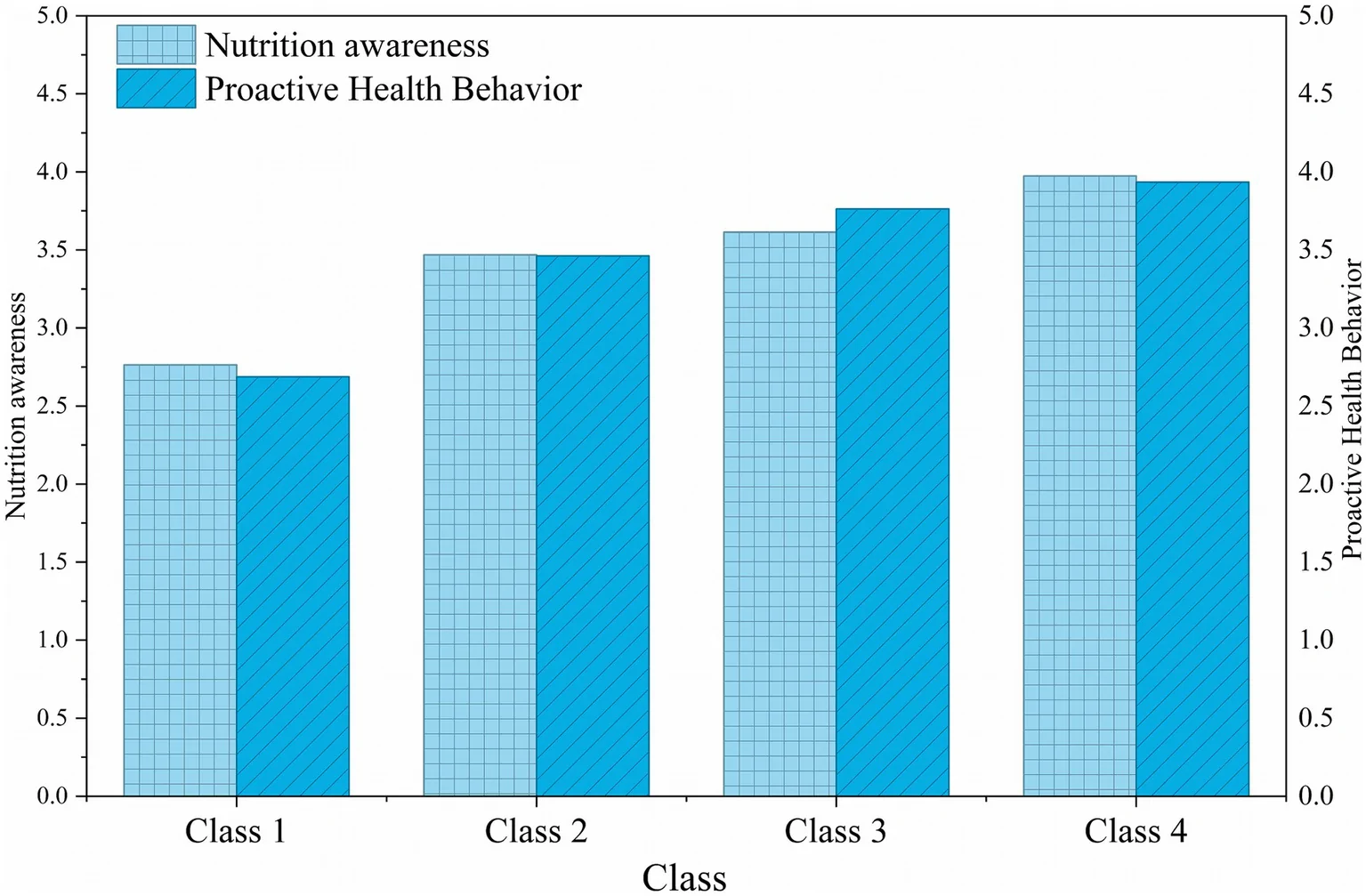
Differences in the optimal potential profile in active health behavior and nutrition awareness.

Notably, the effect size for proactive health behavior across profiles (η^2^ = 0.364) was larger than that for nutrition awareness (η^2^ = 0.271), suggesting that food and nutrition literacy profiles had a more pronounced discriminative effect at the behavioral level. This finding further complements the variable-centered analysis from a person-centered perspective: food and nutrition literacy can not only linearly predict nutrition awareness and proactive health behavior, but different configurations of food and nutrition literacy characteristics can also classify patients with gastric ulcer into subgroups with significant psycho-behavioral differences.

## 4 Discussion

### 4.1 Variable-centered findings: mechanisms, theoretical integration, and comparison with previous evidence

#### 4.1.1 Theoretical framework and Core findings

The findings revealed that food and nutrition literacy was significantly and positively associated with both nutrition awareness and proactive health behavior, and that nutrition awareness partially cross-sectional mediated the food and nutrition literacy–proactive health behavior relationship. These results are consistent with the core postulates of the information–motivation–behavioral skills (IMB) model (40), which posits that health behavior formation depends on the synergistic operation of health-relevant information, personal motivation, and behavioral skills (41). In the present context, food and nutrition literacy constitutes the informational and skill-based foundation (42), while nutrition awareness aligns with the motivational component, reflecting the degree to which patients subjectively value and attend to nutrition-related health issues (43). The empirical finding that both components were independently associated with proactive health behavior supports the IMB model’s assertion that information and motivation are distinct yet complementary determinants of health behavior.

#### 4.1.2 Pathophysiological mechanisms linking food and nutrition literacy to proactive health behavior in gastric ulcer

To understand why food and nutrition literacy is specifically relevant to proactive health behavior in gastric ulcer patients, it is essential to consider the pathophysiological characteristics of gastric ulcer and their intersection with dietary behavior. The gastric mucosa is protected by a multi-layered defense system comprising the mucus-bicarbonate barrier, mucosal blood flow, prostaglandin-mediated cytoprotection, and epithelial restitution mechanisms (44).

Patients with higher food and nutrition literacy are better equipped to recognize these diet–mucosa interactions and translate this understanding into concrete dietary modifications, such as avoiding known trigger foods, maintaining regular meal patterns, reducing sodium intake, and ensuring adequate intake of gastroprotective nutrients (45). This knowledge-to-action translation represents the direct pathway through which food and nutrition literacy facilitates proactive health behavior. Furthermore, emerging evidence on the gut–brain axis suggests that chronic gastric inflammation may influence dietary behavior through neuroimmune pathways (46). Pro-inflammatory cytokines released during active ulceration can alter hypothalamic–pituitary–adrenal axis function and neurotransmitter metabolism, potentially affecting food reward processing, appetite regulation, and stress-induced eating patterns (47). Higher food and nutrition literacy may equip patients with the cognitive resources to recognize and counteract these biologically driven dietary impulses through conscious self-regulation, providing an additional mechanistic pathway through which food and nutrition literacy supports proactive health behavior in this population.

#### 4.1.3 The cognitive–motivational–behavioral Cascade: indirect pathway

The indirect pathway through nutrition awareness reveals a cognitive–motivational–behavioral cascade that extends the simple knowledge–behavior model (48). Drawing on the Health Action Process Approach (HAPA) (49), the formation of health behavior can be decomposed into a motivational phase and a volitional phase (50). Food and nutrition literacy primarily operates in the pre-intentional phase by providing the informational substrate for risk perception and outcome expectancies, whereas nutrition awareness bridges the pre-intentional and intentional phases by consolidating these cognitions into a coherent motivational orientation. Specifically, patients with higher food and nutrition literacy are more likely to: (a) accurately perceive the personal relevance of nutrition information to their disease management; (b) develop positive outcome expectations regarding the benefits of dietary adherence; and (c) build confidence in their ability to implement dietary changes.

#### 4.1.4 Critical interpretation of the direct effect dominance

A notable finding of this study is that the direct effect of food and nutrition literacy on proactive health behavior accounted for 78.58% of the total effect, substantially exceeding the indirect effect through nutrition awareness (21.42%). Unlike purely declarative knowledge constructs, food and nutrition literacy encompasses procedural and behavioral skills that may directly enable health behavior without requiring additional motivational mediation (25). A patient who knows how to prepare a low-fat, non-irritating meal can enact this behavior immediately upon deciding to do so, whereas a patient who knows that such a diet is beneficial but lacks practical skills may still fail to act despite high motivation. What is more, in chronic disease management, dietary behaviors that are initially motivation-dependent can, through repetition and contextual cuing, become habitual and relatively automatic. Patients who have managed gastric ulcer for extended periods may have developed stable dietary routines that are triggered by situational cues rather than by conscious deliberation about nutrition (51). In such cases, the motivational pathway through nutrition awareness becomes less relevant to ongoing behavior, even though food and nutrition literacy continues to show a statistical association with behavior.

### 4.2 Person-centered analysis: heterogeneity, profile characteristics, and added value

#### 4.2.1 Latent profile structure and interpretation

From a person-centered perspective, LPA identified four distinct classes of food and nutrition literacy among gastric ulcer patients: the low food and nutrition literacy group (Class 1, 10.29%), the moderately low food and nutrition literacy group (Class 2, 10.77%), the moderately high food and nutrition literacy group (Class 3, 8.61%), and the high food and nutrition literacy group (Class 4, 70.34%). The model demonstrated excellent classification quality, indicating that patients within each class showed highly homogeneous food and nutrition literacy profiles that were clearly differentiated from those in other classes.

#### 4.2.2 Specific subgroup analysis

The finding that over 70% of the sample was classified into the high food and nutrition literacy group is a striking feature of our results that warrants careful interpretation.

All participants were recruited from the gastroenterology departments of two tertiary Grade A hospitals in Zhejiang Province. Patients who attend tertiary hospitals for gastric ulcer management represent a selected subpopulation that differs systematically from the broader gastric ulcer population in China. Specifically, tertiary hospital patients are more likely to: have been exposed to repeated health education during follow-up visits; have higher baseline health literacy due to the self-selection involved in seeking care at advanced medical facilities; and have better access to health information resources. These factors would be expected to produce an upward shift in the food and nutrition literacy distribution relative to the full gastric ulcer population.

The food and nutrition literacy Scale relies on self-reported agreement with statements describing nutrition knowledge, skills, and behaviors. In a clinical setting, social desirability pressures may lead patients to overestimate their nutrition competencies to align with perceived expectations of the “good patient” role (52). This tendency may be particularly pronounced in Chinese cultural contexts, where deference to medical authority and the desire to present oneself favorably to healthcare providers are well-documented social norms (53). If social desirability bias systematically inflated food and nutrition literacy scores in our sample, the threshold for classification into the “high” class would be artificially lowered, potentially shifting patients from the moderately high to the high food and nutrition literacy class and contributing to the observed concentration in Class 4. We recommend that future studies incorporate objective, performance-based nutrition literacy assessments alongside self-report measures to disentangle true competence from perceived competence and to examine whether the LPA class structure differs when objective indicators are used.

#### 4.2.3 Methodological contributions from an individual centered perspective

A central contribution of this study is the demonstration that person-centered and variable-centered approaches provide complementary, no-redundant insights into the food and nutrition literacy–nutrition awareness–proactive health behavior nexus.

In variable-centered mediation model, this average effect conceals substantial heterogeneity: the four LPA classes differed significantly in both nutrition awareness and proactive health behavior. These effect sizes far exceed the variance explained by any single demographic variable in this study and demonstrate that aggregating across latent classes. The person-centered approach brings this variation into focus, revealing that the “average” patient described by regression coefficients does not correspond to any actual subgroup of gastric ulcer patients, each of whom has a specific food and nutrition literacy profile with specific implications for their nutrition awareness and behavior.

The variable-centered analysis identifies food and nutrition literacy, nutrition awareness, and income as continuous predictors of proactive health behavior, but does not specify whom to target or what to target for maximal intervention efficiency. The person-centered analysis identifies four discrete subgroups with qualitatively distinct food and nutrition literacy configurations, enabling: (1) risk stratification based on profile membership; (2) matching of intervention intensity and modality to profile characteristics; and (3) efficient resource allocation by concentrating intensive, expensive interventions on the highest-risk subgroups while providing lighter-touch maintenance support to the majority. This triage logic cannot be derived from variable-centered regression coefficients.

The variable-centered approach treats each food and nutrition literacy dimension as an independent contributor to outcomes. However, LPA captures the configuration of dimension scores, which may carry predictive information beyond the dimension scores themselves. For example, although Classes 2 and 3 appear symmetric in their naming, their profile shapes may reveal differential patterns of deficit that would require qualitatively different interventions. Variable-centered analyses cannot reveal these configural patterns because they examine each dimension independently.

### 4.3 Clinical and practical implications

#### 4.3.1 Rationale for profile-based stratified intervention

This study provides both empirical evidence and a conceptual framework to support the integration of food and nutrition literacy assessment and profile-based stratification into the clinical management of gastric ulcer. The variable-centered findings establish that food and nutrition literacy and nutrition awareness are independently associated with proactive health behavior and that nutrition awareness constitutes a viable intervention target. The person-centered findings add that patients are not uniformly positioned along these dimensions and that intervention needs differ qualitatively across food and nutrition literacy profiles. The 20-item food and nutrition literacy Scale can be administered at initial diagnosis, at the first follow-up visit, or during health education sessions. Based on dimension score patterns, patients can be assigned to one of four profiles using the classification criteria established in this study.

#### 4.3.2 Profile-specific intervention framework

Building on the characteristic patterns of each latent profile, we propose a tiered intervention framework that matches intervention intensity, modality, and content to profile membership.

High food and nutrition literacy group: Maintenance and Peer Leadership. Patients in this group have adequate food and nutrition literacy, nutrition awareness, and proactive health behavior, representing an asset rather than a liability for the healthcare system. The intervention goal is sustainability and capitalizing on their capabilities. Recommended strategies include: (a) low-frequency maintenance contact (e.g., quarterly newsletters with updated nutrition information, seasonal dietary recommendations); (b) integration into shared medical appointments where they can model effective self-management; and (c) periodic re-screening to detect patients whose food and nutrition literacy or behavior may decline due to life stressors, comorbid conditions, or waning motivation. These patients can be managed with minimal additional clinical resources, freeing capacity for higher-need groups.

Moderately high food and nutrition literacy group: motivational activation. These patients possess adequate food and nutrition literacy but their nutrition awareness has not kept pace with their knowledge base. The intervention emphasis is motivational. Recommended strategies include: (a) 1–2 sessions of brief motivational interviewing (each 20–30 min) delivered by trained nurses or health educators, focusing on exploring personal values related to health, identifying discrepancies between current dietary behavior and health goals, and eliciting “change talk”; (b) personalized goal-setting using the SMART (Specific, Measurable, Achievable, Relevant, Time-bound) framework, with concrete, near-term dietary goals.

Moderately low food and nutrition literacy group: combined knowledge and motivation building. These patients require parallel strengthening of both cognitive and motivational resources, as they show deficits in food and nutrition literacy without the severe depletion seen in the lowest group. Recommended strategies include: (a) structured group-based nutrition education; (b) practical, hands-on workshops; (c) integration of motivational enhancement techniques within each educational session to ensure that knowledge gains are continuously linked to personal relevance and behavioral intention.

Low food and nutrition literacy group: intensive, multi-component intervention. This group represents the highest clinical priority. These patients face low knowledge, low awareness, and low behavioral engagement, which places them at elevated risk for dietary non-adherence, recurrent ulcer episodes, and potentially serious complications. A multi-component, multi-disciplinary intervention is indicated. Recommended strategies include: (a) individualized nutrition assessment and dietary prescription by a registered dietitian, including a written, simplified meal plan with pictorial support for patients with limited literacy; (b) one-on-one nutrition education delivered in multiple formats and in multiple brief sessions to accommodate limited attention and knowledge retention capacity; (c) involvement of family members or caregivers as active participants in education sessions and as home-based dietary support partners.

#### 4.3.3 Integrating food and nutrition literacy screening into clinical workflow

We propose the following operational pathway for integrating food and nutrition literacy profile-based screening and intervention into routine clinical practice:

Step 1: Initial Assessment. At the time of gastric ulcer diagnosis or at the first follow-up visit after diagnosis, administer the 20-item food and nutrition literacy Scale. Score the scale and classify the patient into one of four food and nutrition literacy profiles using the classification algorithm established in this study.

Step 2: Profile-Matched Triage. Based on profile membership: Class 4 (High food and nutrition literacy): Provide standard dietary education; schedule routine follow-up; re-assess food and nutrition literacy at 6-month intervals. Class 3 (Moderately high food and nutrition literacy): Refer for brief motivational interviewing; schedule follow-up with food and nutrition literacy re-assessment at 3 months. Class 2 (Moderately low food and nutrition literacy): Enroll in group nutrition education program; schedule follow-up with food and nutrition literacy re-assessment at 3 months. Class 1 (Low food and nutrition literacy): Refer for intensive multi-disciplinary intervention; schedule follow-up with food and nutrition literacy re-assessment at 1–3 months.

Step 3: Monitoring and Re-classification. At each follow-up assessment, re-administer the food and nutrition literacy scale. If the patient’s profile has transitioned upward, the intervention tier can be stepped down accordingly. If the profile has not improved or has deteriorated, the intervention should be intensified or modified.

Step 4: Integration with Existing Systems. The food and nutrition literacy screening, classification, and triage algorithm can be integrated into electronic health record systems to minimize clinician burden. Automated scoring and classification with decision support prompts can guide clinicians to the appropriate intervention tier without requiring detailed knowledge of the LPA methodology.

### 4.4 Limitations and future research directions

This study has several limitations that should be carefully considered when interpreting the results and their implications.

First, cross-sectional design and causal inference. This study employed a cross-sectional design, which precludes causal inferences about the relationships among food and nutrition literacy, nutrition awareness, and proactive health behavior. Although we grounded our mediation model in established theoretical frameworks that specify the hypothesized causal ordering, the data are compatible with alternative causal structures. For instance, patients with higher proactive health behavior may seek out more nutrition information and engage more actively with health education, thereby improving their food and nutrition literacy and nutrition awareness. Bidirectional or reciprocal effects are also plausible: as patients successfully implement dietary behaviors, their nutrition awareness may be reinforced through positive feedback, creating a virtuous cycle. Future research should employ longitudinal designs with a minimum of three measurement waves, using cross-lagged panel models or random-intercept cross-lagged panel models to examine within-person temporal dynamics while controlling for stable between-person differences.

Second, reliance on self-report measures. All core variables were assessed using self-report questionnaires. Social desirability bias may lead patients to over-report nutrition knowledge, health-conscious attitudes, and healthy dietary behaviors, potentially inflating absolute score levels and attenuating variance at the upper end of each distribution. Recall bias may affect dietary behavior reports, as patients are asked to aggregate and summarize their typical dietary patterns over unspecified time periods. Although Harman’s single-factor test indicated that common method bias did not pose a severe threat, this test has known limitations and cannot rule out more subtle forms of method variance. To address these measurement concerns, future studies should: (a) incorporate objective, performance-based measures of nutrition literacy alongside self-report scales; (b) employ ecological momentary assessment (EMA) via smartphone applications to capture dietary behavior in real time and in natural contexts, reducing retrospective recall demands.

Third, participants were recruited using convenience sampling. Unlike probability-based sampling, convenience sampling may systematically over-represent patients who are: (a) more accessible to researchers; (b) more willing to participate in research; and (c) healthier and more physically capable of completing a questionnaire. These selection factors may bias the estimated prevalence of food and nutrition literacy profiles and may also affect the estimated strength of associations among variables. The net direction and magnitude of these selection biases are difficult to estimate, but they collectively suggest that the findings may not generalize to the full clinical spectrum of gastric ulcer patients.

Fourth, all participants were recruited from the gastroenterology departments of only two tertiary Grade A hospitals, both located in Zhejiang Province, a relatively developed coastal province in eastern China. This narrow geographic and institutional scope limits generalizability in at least three respects. (1) Tertiary hospitals serve as referral centers and manage more complex cases; patients in primary and secondary care settings may differ in disease severity, comorbidity burden, healthcare access, and health literacy. (2) Geographic representation: Zhejiang Province ranks among the top provinces in China in terms of economic development, educational attainment, and health literacy. (3) Cultural context: Dietary culture, health beliefs, and healthcare-seeking norms in Zhejiang may differ from other regions of China and certainly from other countries, limiting the cross-cultural applicability of our findings. Multi-center studies spanning multiple provinces, hospital levels, and culturally diverse regions are needed to establish the generalizability of the four-profile classification and the mediation model.

Fifth, while the four-class solution demonstrated excellent statistical fit and classification quality in our sample, several methodological caveats apply to the interpretation and generalization of LPA results. (a) LPA class enumeration is influenced by sample size, indicator distributional properties, class separation, and model specifications; different samples—even from nominally similar populations—may yield different numbers or shapes of profiles. (b) The selection of classification indicators constrains the possible profile structures; different indicator sets could produce different classifications. (c) LPA assumes that latent classes are discrete categories, whereas the underlying food and nutrition literacy distribution may be continuous or quasi-continuous. We recommend that future studies: perform formal multi-sample cross-validation with holdout samples; conduct sensitivity analyses varying the indicator set to assess the robustness of the class structure; compare LPA with factor mixture models and latent class factor analysis to evaluate the assumption of discrete classes; and, where longitudinal data are available, employ latent transition analysis to examine the stability of profile membership over time and to identify predictors of transition between profiles.

## 5 Conclusion

Based on cross-sectional survey data, this study systematically examined the relationships among food and nutrition literacy, nutrition awareness, and proactive health behavior in patients with gastric ulcer from two complementary perspectives: variable-centered and person-centered approaches. The findings further confirmed that nutrition awareness exerted a significant cross-sectional partial mediating effect between food and nutrition literacy and proactive health behavior. Latent profile analysis further revealed that patients with gastric ulcer could be classified into four latent classes: the low food and nutrition literacy group, the relatively low food and nutrition literacy group, the relatively high food and nutrition literacy group, and the high food and nutrition literacy group. This study provides relatively clear practical implications for the long-term management of patients with gastric ulcer. In clinical nursing and health management practice, food and nutrition literacy assessment should be incorporated into routine follow-up or health education procedures, so as to identify patients with insufficient nutrition literacy, weak nutrition awareness, and low proactive health behavior at an early stage. Meanwhile, stratified interventions should be implemented according to patients’ latent class membership. However, because this study adopted a cross-sectional design, strict causal inferences regarding the relationships among variables cannot be made. Future studies may employ longitudinal follow-up, multicenter sampling, and intervention study designs to further examine the temporal sequence and dynamic mechanisms among food and nutrition literacy, nutrition awareness, and proactive health behavior.

## Data Availability

The raw data supporting the conclusions of this article will be made available by the authors, without undue reservation.
